# The RACOON viral pneumonia score for structured reporting of pre-existing, acute, and post-pneumonic findings on chest CT

**DOI:** 10.3389/fmed.2025.1578282

**Published:** 2025-07-23

**Authors:** Thorsten Persigehl, Philipp Fervers, Andreas Bucher, Peter Isfort, David Maintz, Tobias Penzkofer, Marwin Sähn, Thomas Vogl, Jonathan Kottlors, Felix Doellinger

**Affiliations:** ^1^Institute for Diagnostic and Interventional Radiology, University Hospital Cologne, University of Cologne, Cologne, Germany; ^2^Department of Diagnostic and Interventional Radiology, Goethe University Frankfurt, Frankfurt am Main, Germany; ^3^Department of Radiology, University Hospital Aachen, Aachen, Germany; ^4^Department of Radiology, Hospital Friederikenstift, Hannover, Germany; ^5^Department of Radiology, Charité - Universitätsmedizin Berlin, Berlin, Germany

**Keywords:** computed tomography, COVID-19, pandemic preparedness, pneumonia, structured reporting

## Abstract

**Background:**

The multi-dimensional RACOON Viral Pneumonia Score (RVPS) was developed to compensate for the main weaknesses of the established one-dimensional chest computed tomography (CT) scores. It aimed to quantify the severity of pneumonia and qualitatively monitor infectious lung disease from the acute stage to post-pneumonic sequelae.

**Objectives:**

This research focuses on the original development and evaluation of applicability and inter-reader reliability of the RVPS.

**Methods:**

Within the Radiological Cooperative Network (RACOON), the scoring system was developed after several expert meetings and tested in this proof-of-concept study with 8,525 observations. In the subset of inter-reader validation (7,800 observations), eight blinded radiologists applied the RVPS and evaluated the following CT findings for each lung lobe individually: (I) pure ground glass opacities (GGO), (II) GGO and interstitial thickening, (III) consolidations, (IV) linear opacities and reticulations, and (V) fibrotic-like changes. The extent of each pathology was scored on a scale of 0–5 points, and the total involvement was calculated. Inter-reader variability was assessed using Kendall’s W.

**Results:**

Overall inter-reader reliability of the RVPS was excellent (Kendalls’ W 0.95). CT findings associated with acute pneumonia were scored with good agreement (W 0.81-0.87). Moderate uncertainty was introduced when separating category IV vs. category V findings (W 0.55-0.69). The overall extent of post-infectious findings was assessed with good agreement (W 0.79). The longitudinal distribution of subscores allowed for differentiation between acute pneumonia and post-pneumonic sequelae.

**Conclusion:**

This study presents the RVPS as a comprehensive tool for inter-reader reliable evaluation, longitudinal monitoring, and structured documentation of the extent as well as quality of chest CT findings in infectious lung disease.

## Highlights

- The Racoon Viral Pneumonia Score (RVPS) is a multidimensional instrument for structured, quantitative, and qualitative assessment of (post-)pneumonia CT findings.
- The RVPS proved robust in multi-center inter-reader evaluation.
- The RVPS may facilitate the application of artificial intelligence (AI) and represents a tool for pandemic preparedness.The RVPS may facilitate the application of artificial intelligence (AI) and represents a tool for pandemic preparedness.

## Introduction

Computed tomography (CT) plays a key role in the differential diagnosis of pulmonary infection, as well as the assessment of disease severity and the monitoring of recovery (1, 2). As a most recent example, a broad number of international publications have reviewed the characteristic imaging findings of COVID-19 pneumonia and their typical time course (1, 3–6). In the early stage, the CT shows pure ground glass opacities (GGO) (3, 5). In progressive pneumonia, interstitial septal thickening is visible, followed by consolidations in the peak stage (3, 5). During clinical recovery, GGO and consolidations may persist for several weeks, and potentially reversible thickening of the pulmonary interstitium might still be apparent. Irreversible sequelae include fibrotic-like findings, e.g., chronic partial atelectasis, scarring architectural distortion, and irreversible honeycombing, which were found in up to 10% of discharged COVID-19 patients after 6 months (7, 8). Similar stages have been described for other atypical pneumonias, such as Influenza virus (9). Respiratory viruses are commonly considered to carry the highest potential of causing upcoming pandemics, which underlines the role of chest CT in pandemic preparedness (10).

Identification of the pneumonia-causing pathogen based on CT imaging is a difficult undertaking, as pathognomonic findings are virtually non-existent. However, typical patterns and combinations of findings might specify the differential diagnosis (11). E.g., consolidation in lobar distribution is suggestive of bacterial pneumonia, whereas a predominance of GGO is suggestive of viral pneumonia (11, 12). In some cases, the CT may be more specific for a particular infectious agent, such as bilateral GGO with subpleural sparing in pneumocystis pneumonia (13). In addition to accurate chest CT reporting, the differential diagnosis must be made in a multidisciplinary approach, considering the epidemiologic, clinical, and laboratory setting (11). Besides the qualitative classification of pneumonia-related lung changes, chest CT also shows the quantitative dimension of lung involvement and thus allows for conclusions about the individual disease severity (3).

During the COVID-19 pandemic, various visual scoring systems for chest CTs were published to quantify the extent of lung involvement (14–17). From the authors’ point of view, the “Pan Score” is the most established worldwide (3, 14). Each of the five lung lobes is assigned a score between 0 and 5, with 0 representing “no involvement” and 5 representing “>75% involvement” of the volume of the assessed lobe. “Involvement” includes every finding that can occur in the context of an acute lung infection; accordingly, no distinction is made between, for example, GGO and consolidations. The total score is the sum of all lung lobes and can reach a value between 0 and 25 (3).

In principle, the Pan Score is applicable to pneumonias caused by pathogens other than COVID-19, but it was not developed for this purpose and, to the best of our knowledge, has not been convincingly validated. The herein presented RACOON Viral Pneumonia Score (RVPS) is intended to address two critical weaknesses of the Pan Score and comparable systems while remaining easy to apply: First, the RVPS should be applicable to any conceivable viral pneumonia; Secondly, the RVPS should add a qualitative dimension to visual CT scoring, distinguishing various infectious disease-related CT patterns and preexisting pulmonary changes that are not recognized by the Pan Score. In short, the present study introduces the RVPS as a novel visual scoring system that provides qualitative and quantitative information about infectious and post-infectious chest CT findings. This original RVPS study focusses on the assessment of inter-reader reliability to ensure generalizability of our method.

To validate such a pneumonia scoring system, a large-scale, well-structured database of imaging data is needed. Such an opportunity is offered by the RAdiological COOperative Network (RACOON), as part of the national university medicine network (NUM) founded by the German Federal Ministry of Education and Research (BMBF) in 2020. Within the RACOON network of 36 university hospitals and associated cooperation partners, a comprehensive structured database of pulmonary findings in chest CT and corresponding clinical parameters was established. This unique research infrastructure allows for the detection of clusters of specific imaging findings, which RACOON could monitor in real-time on a nationwide scale. In this way, the RACOON network is expected to play a key role in Germany’s pandemic preparedness program (18). The RVPS is designed as the central RACOON documentation tool for structured reporting of infectious lung disease with the potential for later automation by AI.

## Materials and methods

### Development and design of the RVPS

The RVPS was developed in several expert meetings and discussed on exemplary cases from February 2022 to December 2023.

**Figure 1:**
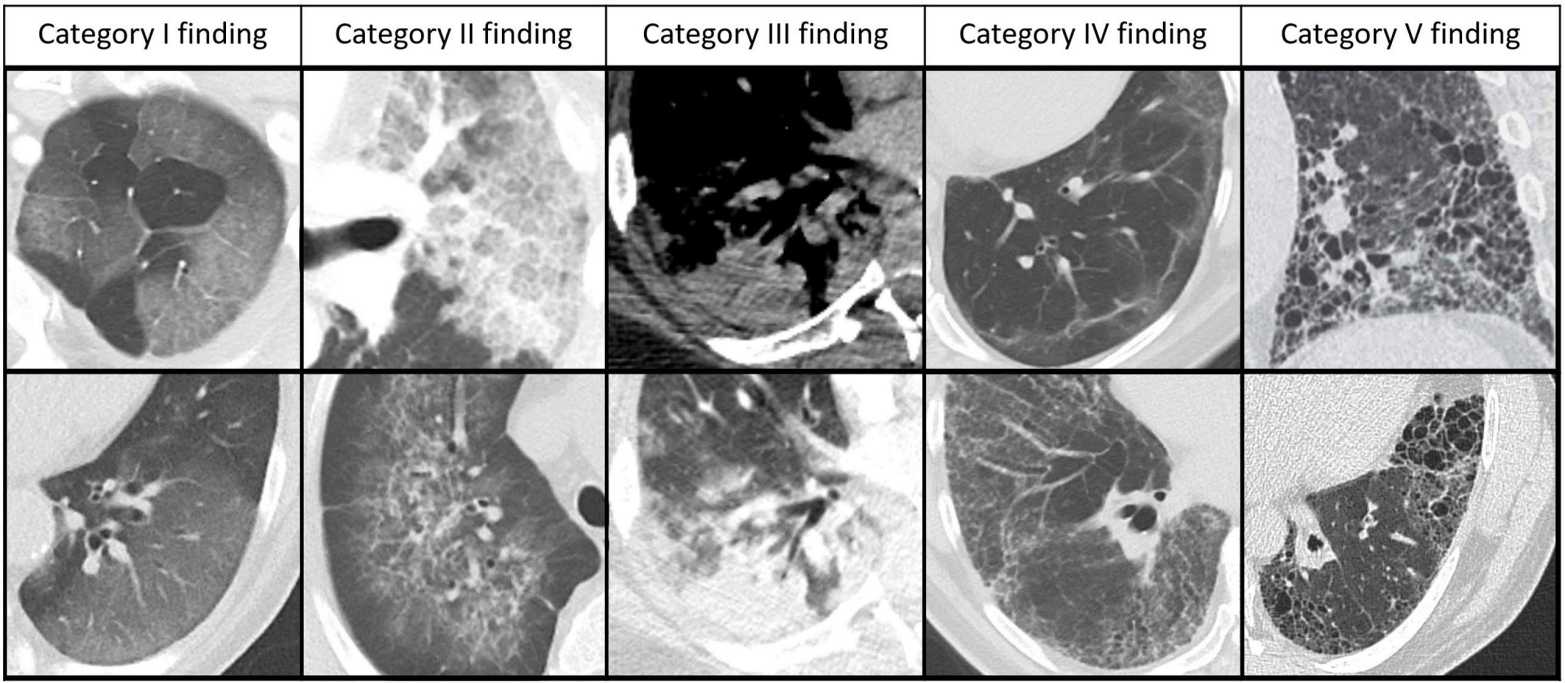
Chest CT findings categories of the RACOON Viral Pneumonia Score. This figure shows five different CT findings categories, which are in principle non-specific but have been described consecutively in the staged course of COVID-19 pneumonia and are therefore subscores of the RACOON Viral Pneumonia Score (RVPS). From left to right: Category I findings comprise pure ground glass opacities (GGO). Category II findings comprise GGO superimposed by interstitial septal thickening, which may result in a crazy paving pattern. Category III findings comprise consolidations, which are often surrounded by GGO. Category IV findings comprise diverse, potentially reversible, post-pneumonic findings such as septal lines, parenchymal bands, and reticulations, all of them without traction or architectural distortion. Category V findings comprise fibrotic-like or cystic changes that are considered irreversible sequelae of pneumonia: Reticulations with traction of the surrounding lung parenchyma and bronchi as well as cystic lesions such as honeycombs and pneumatoceles. Bronchus dilatations can be observed in any phase of pneumonia and are therefore not included in the RVPS.

To assess the RVPS from a chest CT, the reader visually estimates the extent of five defined categories of findings. These five categories are based on the established definitions of the Fleischner Society glossary, which is illustrated in Figure 1 (7, 19):

Category I finding: pure ground glass opacities (GGO). The opacity of the lung parenchyma is increased, yet bronchial and vascular margins can still be delineated (19).

Category II finding: GGO with interstitial thickening (19).

Category III finding: consolidation. The lung opacity is increased, obscuring the margins of vessels, airways, and interstitial septa (19).

Category IV finding: lines and reticulations without traction of the surrounding parenchyma, considered potentially reversible (19).

Category V finding: fibrotic-like changes such as coarse reticulations with traction of surrounding lung parenchyma and bronchi. This category also includes thicker-walled cysts, possibly grouped as honeycombing, and thin-walled pneumatoceles, which are considered irreversible (19).

To ensure equal representation of the left and right lung, the RVPS counts the right upper and middle lobes as one. The presence of each of the five categories of findings is verified individually for the four lung sections: (1) right upper lobe + middle lobe, (2) right lower lobe, (3) left upper lobe, and (4) left lower lobe. Analogous to the established Pan Score, each category of findings in each lung section can score 0–5 points, depending on its extent. As with the Pan score, the reviewer must visually estimate what percentage of the volume of the corresponding lung section is involved by the respective finding:

0 points: indicating no involvement,

1 point: 1–5% involvement,

2 points: 6–25% involvement,

3 points: 26–50% involvement,

4 points: 51–75% involvement,

5 points: >75% involvement.

The RVPS is reported using the following format: “overall sum score (category I/category II/category III/category IV/category V scores).” Since several finding categories can be present at the same time, the overall RVPS ranges from 0 to 52 (with a maximum of 4 × 13 per lobe).

### Proof-of-concept of the RVPS based on the RACOON data

For retrospective testing and initial validation of the RVPS, we assessed the RACOON database from two RACOON partners. Collection of the RACOON data was approved by the respective institutional ethics committees, in accordance with the 1964 Helsinki declaration and its later amendments. For proof-of-concept, the RACOON database was screened for exemplary cases of COVID-19 pneumonia with different severity and preexisting lung disease (*n* = 12), as well as non-COVID-19 cases with other viral, bacterial, and pneumocystis jirovecii pneumonia (PCP) (*n* = 9) causes. Inclusion criteria comprised:

- Laboratory-proven infectious lung disease with findings of acute pneumonia in at least one chest CT examination
- At least three consecutive CT examinations contained in the RACOON database
- Timepoint of symptom onset reported in the medical records
- Patient age >18 years.

The RVPS was obtained for each chest CT of the exemplary test patients by radiologists with experience of at least 500 non-COVID and 500 COVID-19 chest CTs. In addition, a subset was independently scored by seven further, equally experienced radiologists to assess inter-reader variability from two RACOON centers. The results were noted in a dedicated Excel sheet containing columns for the RVPS and the Pan Score. Readers 1–4 recorded their time needed to perform each reading. Patient demographics, type of pneumonia infection, and the time after symptom onset were noted from the medical records.

### Morphologic COVID stages

To reflect the typical time course of viral COVID-19 pneumonia, Pan et al. defined four consecutive stages of characteristic chest CT findings: early, progressive, peak, and absorption (3). After frequent reports of post-infectious lung changes in recovered COVID-19 patients, the fifth post-COVID stage was later amended (8). In our study, the stage was classified by an experienced radiologist for each included examination. Readers were blinded to clinical data. To assess inter-reader variability, 20 evaluations were repeated by three further radiologists.

### Statistical analysis

Statistical data analysis was performed using R version 3.6.2 (20). Figures were plotted using the library *ggplot2* (21). Continuous variables were reported as mean and standard deviation. Data was checked for normal distribution by Shapiro–Wilk’s test using the R library *psych* (22). Wilcoxon test was performed to compare non-parametric data, Student’s t-test was performed to compare parametric data.

To assess the extent of concordance of the four individual readers, we tested the inter-reader reliability regarding the individually assigned scores. Due to the ordinal scaled character of the RVPS, we computed the inter-reader reliability with Kendall’s W using the R library *irr* (23). Statistical significance was defined as *p* < 0.05.

## Results

In this study, the readers recorded 8,525 observations in 682 scorings of 68 CT examinations of 21 patients (16 males, age 57.7 ± 13.2 years). Twelve patients suffered from laboratory-proven COVID-19 pneumonia and six patients tested positive for influenza virus. Three further exemplary patients were infected with Pneumocystis jirovecii, *S. pneumoniae*, and respiratory syncytial virus, respectively. Out of the 40 COVID-19 positive CT examinations, the radiologists rated 3 CT scans as stage 1 COVID-19 pneumonia; 12, 11, 8, and 6 CT scans were rated as stages 2 to 5, respectively. The blinded readings resulted in a chronologically correct, increasing order of COVID-19 pneumonia stages for all included patients (e.g., there was no CT scan that was rated stage 1 after stage 2 was already reached).

### Inter-reader reliability of the RVPS

In the inter-reader subset, the eight experienced readers recorded 7,800 observations in 624 scorings of the same 39 CT examinations. Median values of the readings were adopted for further analysis. Inter-reader reliability of the overall Pan Score and RVPS were excellent (Kendall’s W 0.96 and 0.95, respectively). The scoring of the individual extent of infectious chest CT findings, summarized by the categories 1 to 3, namely GGO, GGO with interstitial thickening, and consolidation, indicated very good inter-reader reliability (Kendall’s W 0.81–0.87). The readers were less consistent when scoring the individual extent of the post-infectious findings in category 4 and 5, namely reversible and fibrotic-like changes (Kendall’s W 0.55 and 0.69, moderate). When looking at the sum of each infectious and post-infectious findings, rather than each individual item, inter-reader reliability was excellent/good (Kendall’s W 0.95 and 0.79, respectively).

Inter-reader reliability of stage classification of COVID-19 was very good (Kendall’s W 0.87). Detailed analysis of inter-reader variability is reported in Table 1.

**Table 1:** Inter-reader agreement of the RACOON viral pneumonia score (RVPS) and the Pan Score (PS).

	Category I findings	Category IIfindings	Category III findings	Category IV findings	Category V findings	RVPS	PS
Kendall’s W	0.81	0.86	0.87	0.55	0.69	0.95	0.96
	0.95 (sum of infectious findings, categories 1–3)			0.79 (sum of post-infectious findings, categories 4–5)			

Eight independent radiologists performed the RVPS and PS scorings by recording overall 7,800 observations of 39 CT scans. Category 1 to 3 findings, which are primarily found in acute infectious lung disease, indicated good inter-reader reliability (Kendall’s W = 0.81-0.87, sum of infectious findings excellent Kendall’s W = 0.95). Categories 4 and 5, which represent the post-infectious findings, showed moderate inter-reader reliability (Kendall’s W = 0.55-0.69). The sum of post-infectious findings, however, indicated a good inter-reader reliability (Kendall’s W = 0.79).

### Evaluation of reading time

The mean time taken to yield the RVPS from a single CT examination was 129 ± 40 s. The mean time taken to get the Pan Score was 70 ± 33 s overall. On average, the assessment of the Pan Score was significantly faster than the RVPS scoring, at 59 ± 41 s (Student’s t-test, *p* < 0.05).

### Proof-of-concept: stage-dependent composition of the RVPS in COVID-19 pneumonia

**Figure 2:**
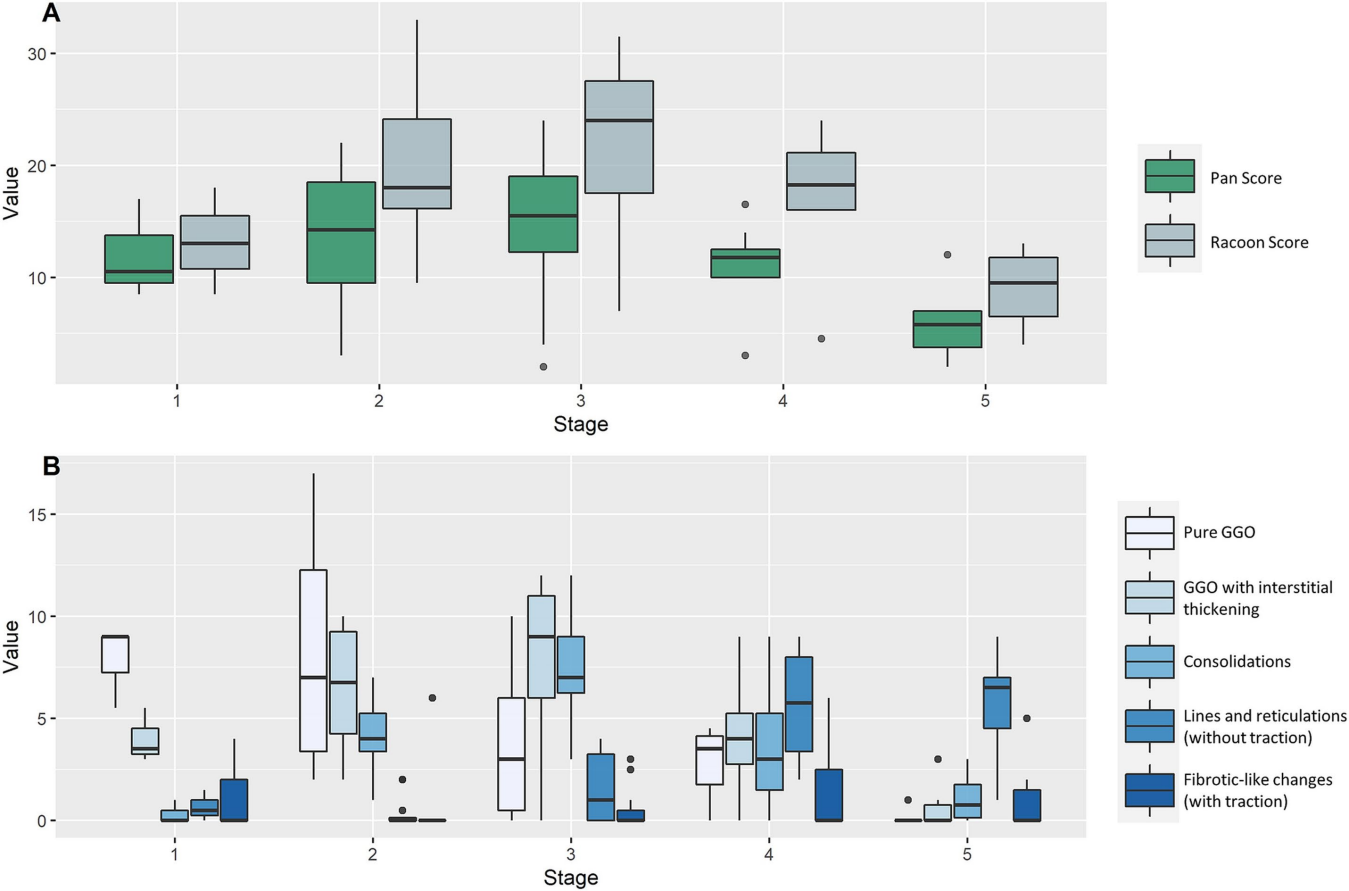
Disease burden and extent of chest CT findings in different stages of COVID-19 pneumonia. Imaging findings of 12 patients with 40 consecutive chest CT examinations are demonstrated as boxplots, grouped by the five COVID-19 pneumonia stages (x-axis). **(A)** Illustrates the extent of lung disease by the Pan Score and RACOON Viral Pneumonia Score (RVPS) as boxplots, which are grouped by the disease stages 1–5. The overall extent of lung disease is most severe during progressive and peak stages (stages 2/3). **(B)** Demonstrates the stage-dependent distribution of the five categories of CT findings: While early and progressive stages are dominated by ground glass opacities (GGO) and interstitial thickening, absorption and post-COVID stages predominantly show linear opacities and fibrotic-like changes. The RVPS is designed to document such stage-specific imaging findings. GGO, ground glass opacities.

**Figure 3:**
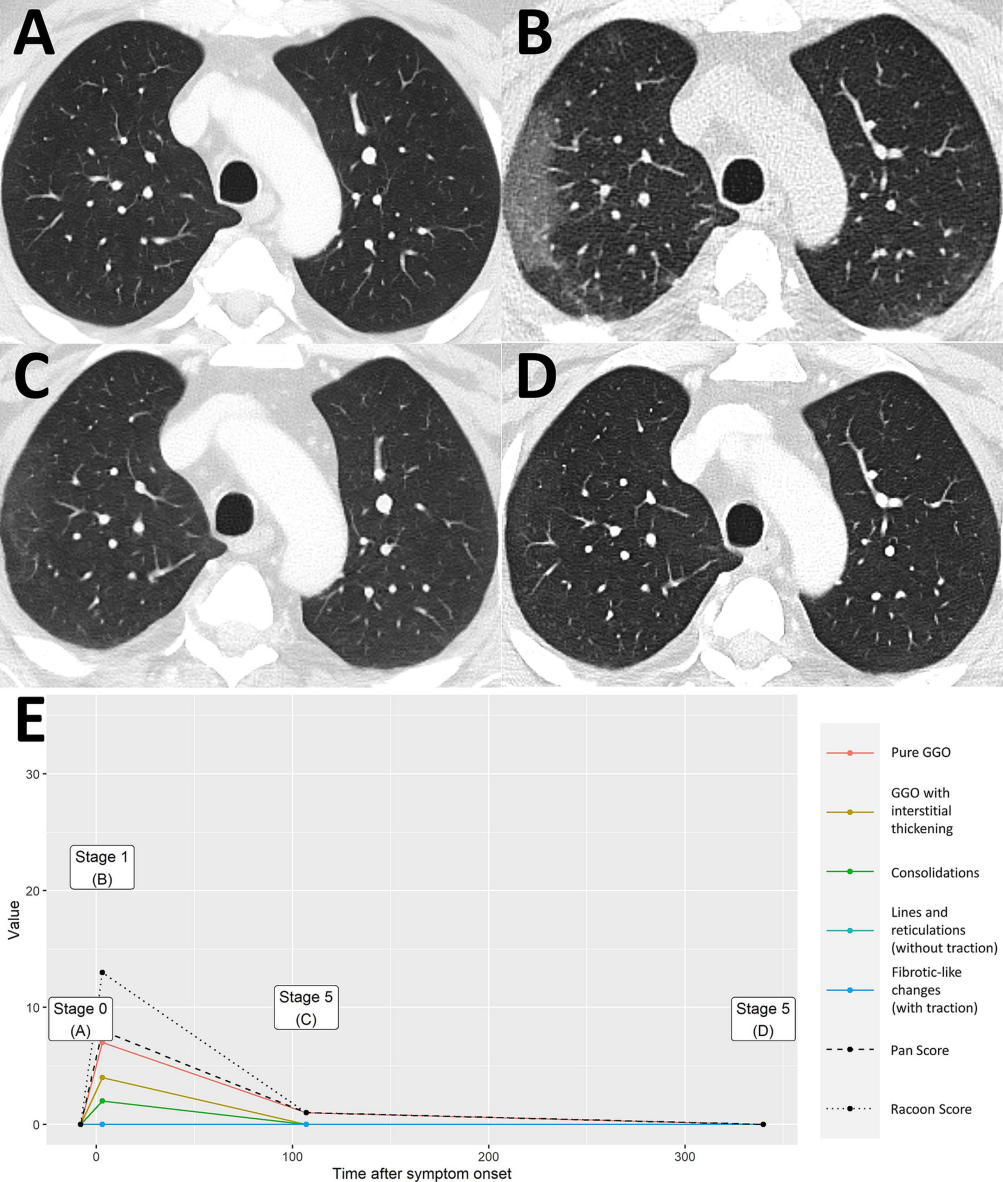
Uncomplicated COVID-19 pneumonia without imaging residuals. A 59-year-old male patient with history of lung cancer. Two weeks after a whole-body imaging for oncological indication **(A)**, he developed fever and mild cough. Chest CT demonstrated typical signs of COVID-19 pneumonia consistent with early stage [**B**, RVPS = 13 (7/4/2/0/0); stage 1]. The two follow-up examinations on day 107 and day 340 after symptom onset were again performed for oncological follow-up and demonstrated imaging recovery of COVID-19 pneumonia [**C**, RVPS = 1 (1/0/0/1/0); stage 4, and **D**, RVPS = 0 (0/0/0/0/0); no perceivable sequelae]. Note that this case serves illustration purposes only. RVPS, RACOON Viral Pneumonia Score; GGO, ground glass opacities.

**Figure 4:**
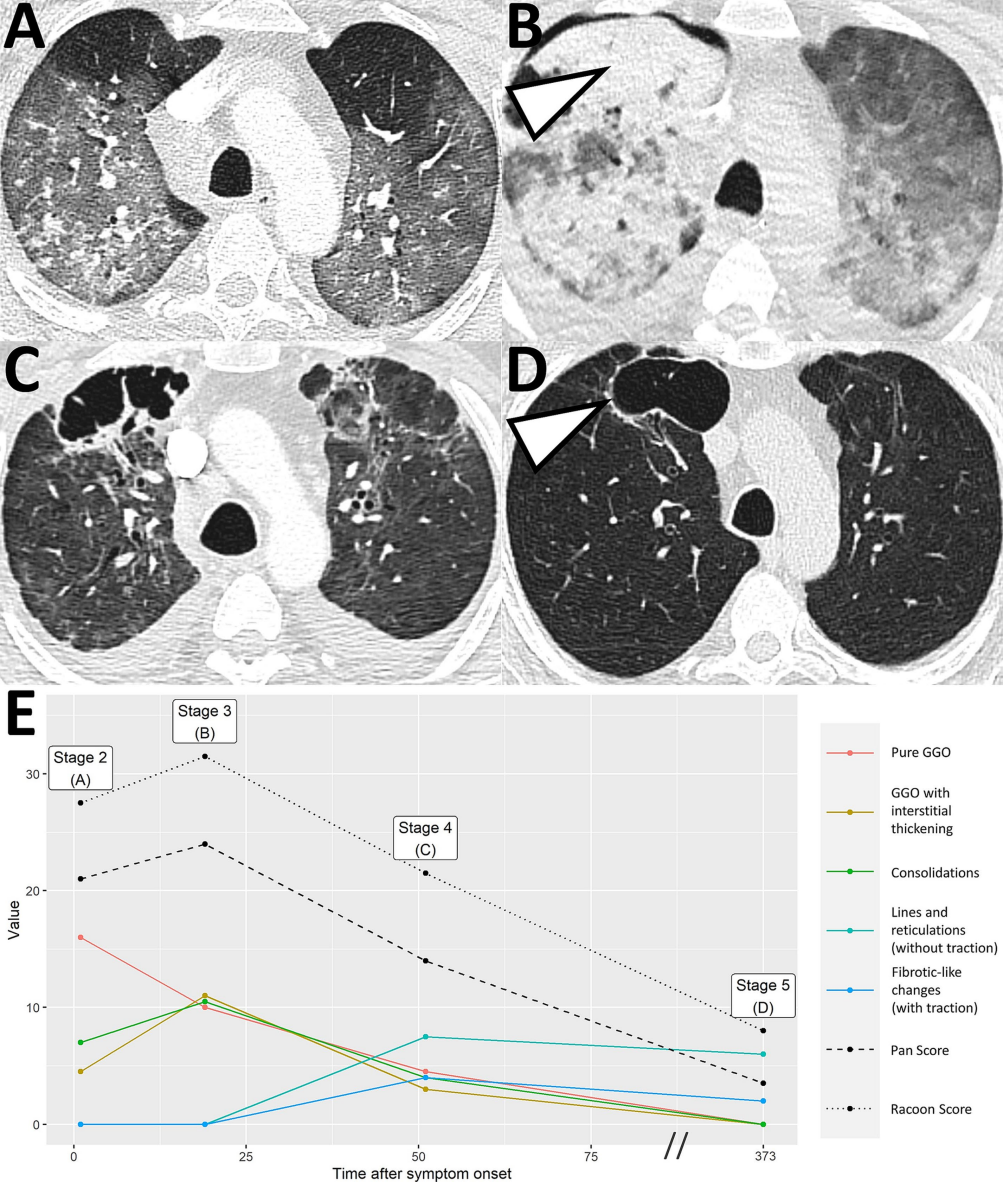
Severe COVID-19 pneumonia with post-COVID-19 sequelae. A 43-year-old male patient presented at the hospital with severe dyspnea 1 day after anamnestic symptom onset of laboratory-proven COVID-19 pneumonia. Initial chest CT demonstrated progressive stage COVID-19 pneumonia with predominantly ground glass opacities and interstitial thickening [**A**, RVPS = 28 (16/5/7/0/0); stage 2]. The second CT scan 19 days after symptom onset showed progression to peak stage findings [**B**, RVPS = 32 (10/11/11/0/0); stage 3]. Extensive consolidation indicated bacterial superinfection, which obligated for intravenous antibiotics. At the location of the dense consolidation in the right upper lobe (white arrowhead in **B**), a third CT scan 51 days after symptom onset reported the formation of a cystic, presumably irreversible lung tissue damage. Yet, there were still findings consistent with infectious pneumonia [**C**, RVPS = 22 (5/3/4/8/4); stage 4]. Follow-up imaging in the post-infectious interval at day 373 after symptom onset confirmed the assumption of an irreversible parenchymal defect [white arrowhead in **D**, RVPS = 8 (0/0/0/6/2); stage 5]. The Pan score and RVPS demonstrate a parallel course **(E)**. However, the RVPS recognizes the formation of post-COVID-19 CT findings (blue line in **E**), which are not appreciated by the Pan Score. RVPS, RACOON Viral Pneumonia Score; GGO, ground glass opacities.

**Figure 5:**
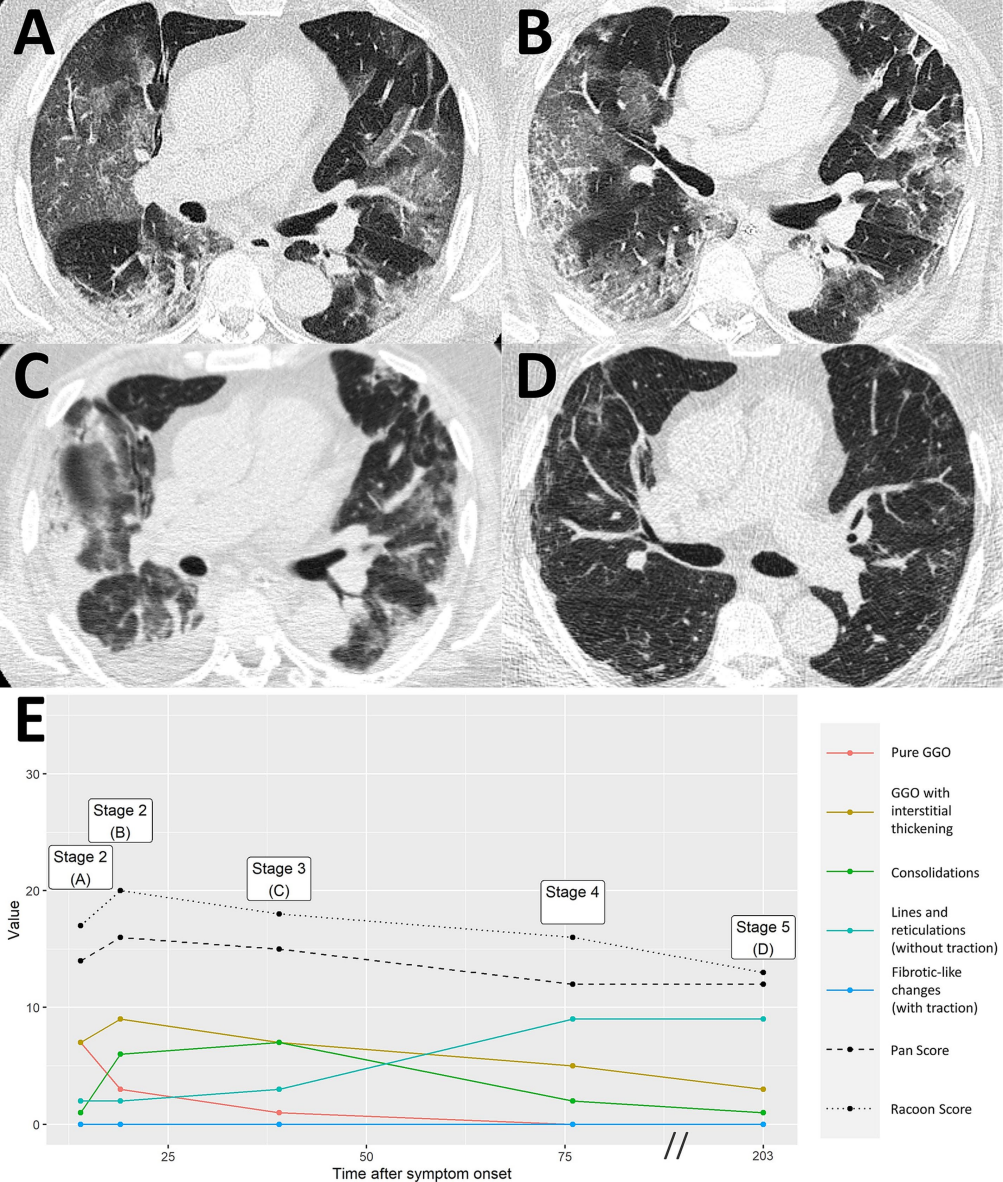
Severe COVID-19 pneumonia with residual imaging findings after >6 months follow-up. A 64-year-old male patient presented at the hospital 14 days after symptom onset, suffering from headaches and difficulty breathing. Chest CT demonstrated extensive ground glass opacities (GGO) with beginning interstitial thickening, consistent with progressive stage COVID-19 pneumonia [**A**, RVPS = 17 (7/7/1/2/0); stage 2]. Another chest CT 19 days after symptom onset reported an advance of the pneumonia to predominantly interstitial pathology and less ground glass [**B**, RVPS = 20 (3/9/6/2/0); stage 2]. Peak stage COVID-19 pneumonia with maximum consolidation was reached at 39 days after symptom onset [**C**, RVPS = 18 (1/7/7/3/0); stage 3]. Follow-up imaging on day 203 after symptom onset showed increasing linear opacities and fine parenchymal bands, yet no fibrotic-like changes with traction [**D**, RVPS = 13 (0/3/1/9/0); stage 5]. The subscores of the RVPS enable appreciation of such a typical time course of COVID-19 pneumonia, which is demonstrated in **(E)**: Early and progressive stages are dominated by GGO and interstitial pathology (red/yellow), followed by a maximum of consolidation in peak stage (green), and eventually formation of linear opacities in resorption and post-COVID-19 stages (turquoise). RVPS, RACOON Viral Pneumonia Score; GGO, ground glass opacities.

**Figure 6:**
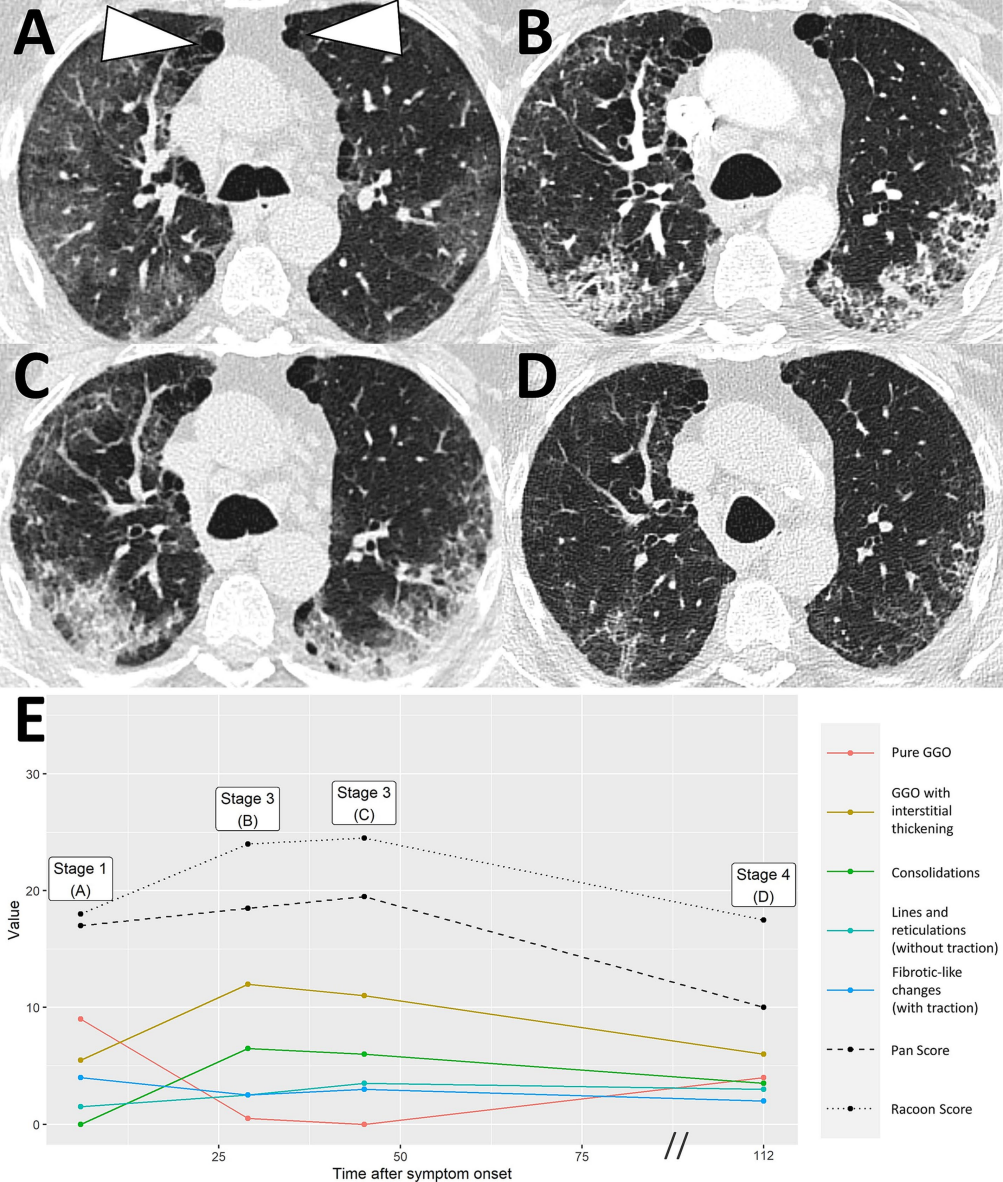
COVID-19 pneumonia in a patient with pre-existing fibrosis. A 66-year-old male patient presented at the hospital 6 days after onset of acute respiratory symptoms. Initial chest CT showed bilateral ground glass opacities (GGO) and paraseptal pulmonary emphysema with minor peripheral fibrosis [white arrowheads, **A**, RVPS = 18 (9/6/0/2/4); stage 1]. The presumably pre-existing pathology remains consistent throughout the otherwise typical staged course of COVID-19 pneumonia with peak stage [**B**, RVPS = 24 (1/12/7/3/3); stage 3, and **C**, RVPS = 25 (0/11/6/4/3); stage 3] and absorption stage [**D**, RVPS = 18 (4/6/4/3/2); stage 4], which is illustrated by the relatively straight blue line in **(E)**. RVPS, RACOON Viral Pneumonia Score; GGO, ground glass opacities.

The overall extent of lung involvement as well as the individual COVID-19-associated chest CT findings varied depending on the stage of the disease. Early, progressive, and peak stages demonstrated mostly ground glass, interstitial thickening, and consolidation. Absorption phase and post-COVID-19 stages were dominated by linear opacities and fibrotic-like changes. Median RVPS for the COVID-19 pneumonia stages 1–5 were 13 (9/4/0/1/0), 18 (7/7/4/0/0), 24 (3/9/7/1/0), 18 (4/4/3/6/0), and 10 (0/0/1/7/0), respectively. The respective Pan Scores were 11, 14, 16, 12, and 6. The detailed distribution of the scores is illustrated in Figure 2. The four following cases demonstrate the clinical use of the RVPS in COVID-19 pneumonia in Figures 3–6. Supplementary Data 1 contains a preliminary decision tree algorithm to perform automated COVID-19 pneumonia staging using the machine-readable RVPS.

### Proof-of-concept: RVPS for documentation of miscellaneous viral, bacterial, and atypical pneumocystis jirovecii pneumonia

**Figure 7:**
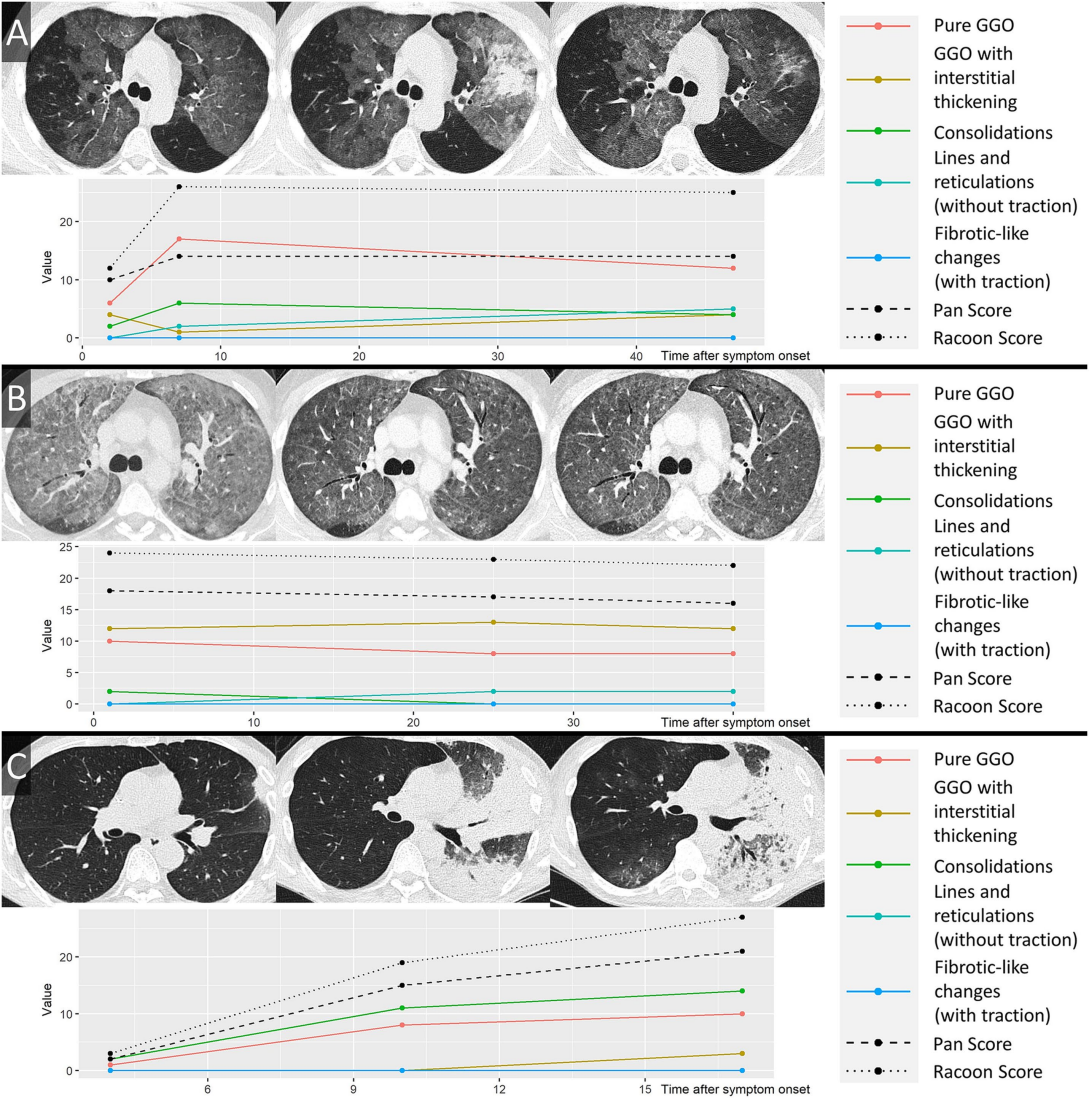
Monitoring of different pneumonia entities by the RACOON Viral Pneumonia Score (RVPS). Three exemplary cases of non-COVID-19 pneumonia are illustrated in **(A–C)** by axial CT slices at three consecutive timepoints each. The RVPS are presented in the corresponding panels using the same format as Figures 3–6 (time after symptom onset vs. value of the RVPS finding). Legends are included on the right side in each panel. **(A)** A 43-year-old male patient presented at the hospital on day 2 after onset of acute respiratory symptoms. Initial chest CT (timepoint 1) showed predominantly ground glass opacifications (GGO), consistent with atypical pneumonia. Virology results confirmed respiratory syncytial virus as the infectious agent. On day 7 after symptom onset (timepoint 2), an increase of consolidation was noted, and antibiotic therapy was required to treat bacterial superinfection. After 47 days (timepoint 3), follow-up CT confirmed decreasing opacification of the lungs. **(B)** A 42-year-old immunocompromised male patient with pneumocystis jirovecii pneumonia (PcP). Timepoints 1–3 on days 1, 25, and 40 after symptom onset demonstrated extensive GGO, consistent with atypical pneumonia. A steady decrease of Pan Score and RVPS illustrated therapy response to antimycotic therapy. **(C)** A 58-year-old male patient with fever and cough for 4 days. Initial chest CT showed a focal consolidation in the left upper lobe (timepoint 1), consistent with bacterial lobar pneumonia. On day 10 after symptom onset (timepoint 2), progression of the pneumonia was apparent by expansion of consolidation to the left lower lobe. The last CT was acquired on day 17 after symptom onset (timepoint 3), showing further advance of the infection with involvement of both lungs. RVPS, RACOON Viral Pneumonia Score; GGO, ground glass opacities.

The RVPS demonstrates a distinct distribution depending on the infectious agent. Pneumocystis jirovecii pneumonia is dominated by category I and II findings (GGO and interstitial thickening), lobar pneumonia shows extensive category III findings (consolidation), and bacterial superinfection of respiratory syncytial virus pneumonia becomes apparent by category I finding (GGO) superimposed with a peak of category III finding (consolidation). Figure 7 illustrates the RVPS on three exemplary non-COVID-19 pneumonia cases.

## Discussion

This study introduces the RACOON Viral Pneumonia Score (RVPS) as a comprehensive and versatile approach for structured CT reporting of infectious and post-infectious lung disease. In the presented study, the five findings categories showed reproducible differences between the different infectious agents as well as the different disease stages of COVID-19 pneumonia. A preliminary, head-to-head comparison showed that the RVPS has merit to disclose the stage of COVID-19 pneumonia when analyzed by a decision tree algorithm. Its accuracy could not be matched by the Pan Score, which is limited by its unidimensional design.

The RVPS is reproducible and robust. In the here presented proof-of-concept study, we could demonstrate the ease of use and ensure excellent generalizability of our method. To prevent misinterpretation of imaging findings, we designed the RVPS as a descriptive instrument. The five findings categories are defined by the established Fleischner glossary, which warrants generalizability for a broader clinical and scientific use (19). The excellent inter-reader reliability suggests reliable data collection for multicenter comparisons, such as within the RACOON network. In contrast to the very good/excellent inter-reader reliability when scoring the acute, infectious chest CT findings (GGO, interstitial thickening, consolidation), the post-infectious findings were scored with only moderate consistency. The arbitrary separation of potentially reversible category 4 findings from presumably irreversible, fibrotic like category 5 findings introduced a moderate degree of uncertainty. This finding is in line with the recent literature. Certain dynamic imaging patterns appear to be associated with irreversible lung damage after viral pneumonia (24). Observational studies with medium-term follow-up have shown that GGO and parenchymal bands resolve more slowly than consolidations or crazy paving, but there are only little long-term data up to now (25). Large-scale studies are urgently needed, as the separation of reversible versus irreversible post-infectious CT findings is not yet fully understood (25, 26). The RVPS serves as a tool to document post-infectious lung disease and might help to raise awareness in the clinical community to appreciate possibly irreversible findings.

Moreover, we propose to use the RVPS in trials for therapy management and prognosis assessment in pneumonia of all types. In one study, it was shown that subtle changes in imaging patterns indicate a response to antiviral therapy in quantitatively stable COVID-19 pneumonia (27). On the other hand, progressive consolidation of the affected lung tissue in COVID-19 pneumonia in combination with neutrophilic leukocytosis indicates toward bacterial superinfection (28). Unidimensional scores, such as the Pan Score, cannot depict such morphological changes if the overall extent of the pneumonia remains the same. Whereas RVPS users can already assess with little practice that an RVPS of 15 (5/9/1/0/0) represents a different clinical situation than an RVPS of 15 (0/0/0/2/13) (in this example, presumably acute viral pneumonia vs. advanced pulmonary fibrosis). This simple example illustrates that the RVPS is universally applicable and not restricted to COVID-19 pneumonia. By applying the RVPS, the reader is encouraged to break down the CT into individual findings, which can help differential diagnosis concerning the various infectious agents.

During the early stages of the pandemic, CT imaging proved an excellent tool to diagnose COVID-19 pneumonia (29). Viruses with transmission through respiratory droplets are currently considered to have the most critical pandemic potential (10). Therefore, it seems plausible that potentially emerging lung diseases could also be differentiated by distinctive imaging patterns. In case of a new pneumonia pandemic that manifests by cavitations, focal calcifications, or tree-in-bud patterns, such findings could be included in a revised version of the modular RVPS, thereby specifying the system for a new disease or pathogen. In a network of cooperating institutions that use the RVPS continuously, the occurrence of new types of infectious diseases is detected by a systematic shift of the subscores toward new combinations. This is a key strength of our score, which promotes frequently demanded pandemic preparedness.

This study has several limitations. One limitation is the narrow and unbalanced patient population, which suggests overfitting of our supplementary staging model. We are very aware that our RVPS algorithm needs to be validated on a larger data set, although this was not the intention of this first proof-of-concept study. The analysis presented here focusses on the development and evaluation of inter-reader reliability of the novel score. The outstanding large-scale validation, multicenter study within the RACOON network is already running. Furthermore, our approach is limited by the known drawbacks of the Pan Score: Due to the overrepresentation of smaller lung infiltrations and the middle lobe of the lung, the score does not show a linear relationship with the quantitatively measured infiltrated lung tissue (14). Although we maintained the established threshold of <5% for 1 point of the RACOON score to appreciate subtle imaging findings, we combined the middle and right upper lobes to improve the balance of our score. To ensure the best inter-reader reliability, the RACOON score captures lung pathologies in only five findings categories defined by the Fleischner Society. Subtle changes in the quality of imaging findings, such as gradually resolving GGO, are not scored if the spatial extent remains constant. This limits the monitoring of minor changes in infectious lung disease by the RVPS. Compared to the Pan Score, scoring the RVPS increased the reading time of human reviewers by approximately 1 min. In a work environment with high time pressure, this might be a relevant limitation. However, we consider this minute to be well invested in order to benefit from the unique possibilities of the RVPS. This additional minute is also put into perspective in view of the total time for the CT reading, including soft tissue and bone window.

In general, the RVPS is machine readable and suitable for automated analyses, as we demonstrated in our supplementary analysis. Recent advances in the field of AI may facilitate automated scoring of the RVPS and weaken the argument of reading time (30).

In summary, with the RVPS, we offer a comprehensive scoring method for standardized, long-term assessment of infectious and post-infectious lung diseases. In this proof-of-concept evaluation, we have proved its reliability in inter-reader settings. Of course, we all hope that this will never happen, but should there ever be another global health emergency like the COVID-19 pandemic, we are optimistic that the RVPS could prove to be a useful tool. In any case, its modular design makes it possible to adapt the combination of selected findings to any pneumonia pathogen that causes typical CT image changes. In this respect, the RACOON Viral Pneumonia Score can certainly be seen as an approach to build resilience against a potential new pandemic. In a subsequent study, we will investigate the multicenter, multivendor, multireader performance of the RVPS within the RACOON-COMBINE network. Future research should focus on potential use cases in monitoring atypical pneumonia, investigating post-infectious lung injury, and assessing treatment response in infectious lung disease.

## Data Availability

The raw data supporting the conclusions of this article will be made available by the authors, without undue reservation.
