# Supplementary material for: The RACOON viral pneumonia score for structured reporting of pre-existing, acute, and post-pneumonic findings on chest CT
Source: Front Med (Lausanne). 2025 Jul 23;12:1578282. doi: 10.3389/fmed.2025.1578282 (PMC12325055; doi:10.3389/fmed.2025.1578282)
Supplement: Supplementary file 1 [file Data_Sheet_1.docx]

**The RACOON Viral Pneumonia Score for structured reporting of pre-existing, acute and post-pneumonic findings on chest CT**

Thorsten Persigehl, Philipp Fervers, RACOON Consortium, Andreas Bucher, Bernd Hamm, Peter Isfort, David Maintz, Tobias Penzkofer, Marwin Sähn, Thomas Vogl, Jonathan Kottlors, Felix Doellinger

**Supplementary material**

**Supplementary Methods**

**Classification of the COVID-19 pneumonia stage by the RACOON viral pneumonia score (RVPS) and the Pan score (PS)**

A decision tree model was fit to perform the classification of COVID-19 pneumonia stage by the RVPS. First, the RVPS was split into its five individual components (finding category I-V scores). Consecutively, the following five input parameters were calculated:

1) the sum of the scores of presumably inflammatory imaging findings (categories I + II + III).

2) - 5) the ratio of finding category I score divided by finding category II score, category II divided by category III, category III divided by category IV, and category IV divided by category V.

The final model contained five decision nodes (Supplementary Figure 1). The model output is summarised in Supplementary Table 1, achieving a classification accuracy of 0.83. The median error of the 9 misclassified cases was 1 [1-1] stage.

The correspondent decision tree model using the Pan score as an input is illustrated in Supplementary Figure 2, achieving an accuracy of 0.53. The median error of misclassified cases was 2 [1-2] stages. The classification performance by the Pan score is summarized in Supplementary Table 2.

Supplementary analysis was performed to compare the COVID-19 stage classification performance of the RVPS vs. the Pan score. Hereby, the RVPS achieved a more accurate and more reliable COVID-19 pneumonia stage classification compared to the widely used Pan score (accuracy of 0.83 vs. 0.53 and median classification error of 1 [1-1] vs. 2 [1-2] stages, respectively). Since the decision tree models were calculated retrospectively and on a dataset of only 40 CTs, they might be subject to overfitting and should not be used in clinical decision making in their current form. The large-scale validation study is already projected within the RACOON consortium. Yet, this preliminary supplementary analysis shows that the RVPS has merit to serve as a structured, machine-readable classification tool of infectious lung disease. On the other hand, the one-dimensional Pan score was not adequately responsive to qualitative changes of COVID-19 pneumonia.

**Supplementary Figures:**

Supplementary Figure 1: Decision tree algorithm to classify the COVID-19 pneumonia stage by the RACOON score.

A decision tree model was fit to perform staging of COVID-19 pneumonia, requiring the RACOON score as its input parameter. First, the five following items were calculated from the RACOON score:

Cat I + II + III: Absolute sum of the presumably inflammatory findings, including imaging categories I, II, and III.
Cat I / II, Cat II / III, Cat III / IV, and Cat IV / V: The ratios of the scores of consecutive imaging finding categories.

Consecutively, the data was split by five arbitrary decision nodes, which achieved a retrospective classification accuracy of 0.83. Each node is labelled with three attributes: First, the predicted class (stage 1-5). Second, the probability of each class, concerning the observations in the respective node. A darker shade of colour of a node implies a more certain classification. Third, the percentage of observations in the node (the model required at least 10% of observations to create a new node). Note that the item “category IV / V” was not adopted in the final model.

Supplementary Figure 2: Decision tree algorithm to classify the COVID pneumonia stage by the Pan Score.

Classification of the COVID pneumonia stage by the Pan Score required four arbitrary decision nodes. The model’s accuracy achieved 0.53. COVID stage 1 could not be successfully classified. Each node is labelled with three attributes: First, the predicted class (stage 1-5). Second, the probability of each class, concerning the observations in the respective node. A darker shade of colour of a node implies a more certain classification. Third, the percentage of observations in the node (the model required at least 10% of observations to create a new node).

**Supplementary Tables:**

Supplementary Table 1: Decision tree model performance for COVID-19 pneumonia staging by the RACOON Score.

| actual →  predictions ↓ | Stage 1 | Stage 2 | Stage 3 | Stage 4 | Stage 5 |
| --- | --- | --- | --- | --- | --- |
| Stage 1 | 3 | 2 | 1 | 0 | 0 |
| Stage 2 | 0 | 8 | 1 | 0 | 0 |
| Stage 3 | 0 | 2 | 9 | 0 | 0 |
| Stage 4 | 0 | 0 | 0 | 7 | 0 |
| Stage 5 | 0 | 0 | 0 | 1 | 6 |

Out of 40 classifications, 33 (0.83) were performed correctly. The predicted stage of the 7 misclassified cases mostly failed by only 1 stage; merely 1 case was misclassified by 2 stages (actual: stage 3, predicted: stage 1).

Supplementary Table 2: Decision tree model performance for COVID-19 pneumonia staging by the Pan score.

| actual →  predictions ↓ | Stage 1 | Stage 2 | Stage 3 | Stage 4 | Stage 5 |
| --- | --- | --- | --- | --- | --- |
| Stage 1 | 0 | 0 | 0 | 0 | 0 |
| Stage 2 | 0 | 6 | 3 | 1 | 0 |
| Stage 3 | 1 | 2 | 5 | 1 | 0 |
| Stage 4 | 2 | 1 | 1 | 5 | 1 |
| Stage 5 | 0 | 3 | 2 | 1 | 5 |

Out of 40 CT scans, the stage was classified correctly in 21 cases (accuracy 0.53). The 19 misclassified cases included 9 misclassifications by 1 stage, 5 misclassifications by 2 stages, and 5 misclassifications by 3 stages.
