# Supplementary figures and images for: The RACOON viral pneumonia score for structured reporting of pre-existing, acute, and post-pneumonic findings on chest CT
Source: Front Med (Lausanne). 2025 Jul 23;12:1578282. doi: 10.3389/fmed.2025.1578282 (PMC12325055; doi:10.3389/fmed.2025.1578282)

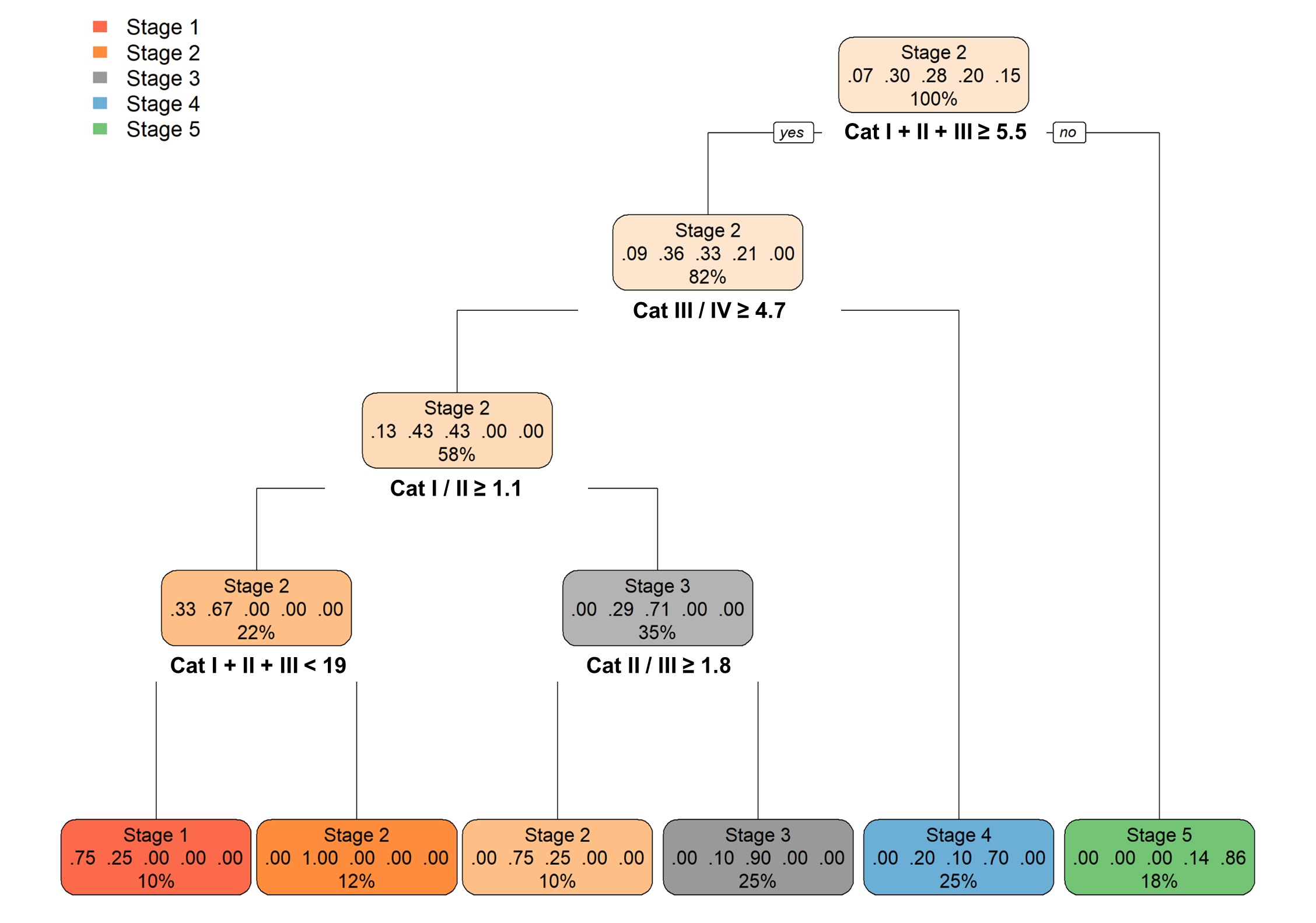

Supplement: Supplementary file 2 [file Image_1.jpeg]

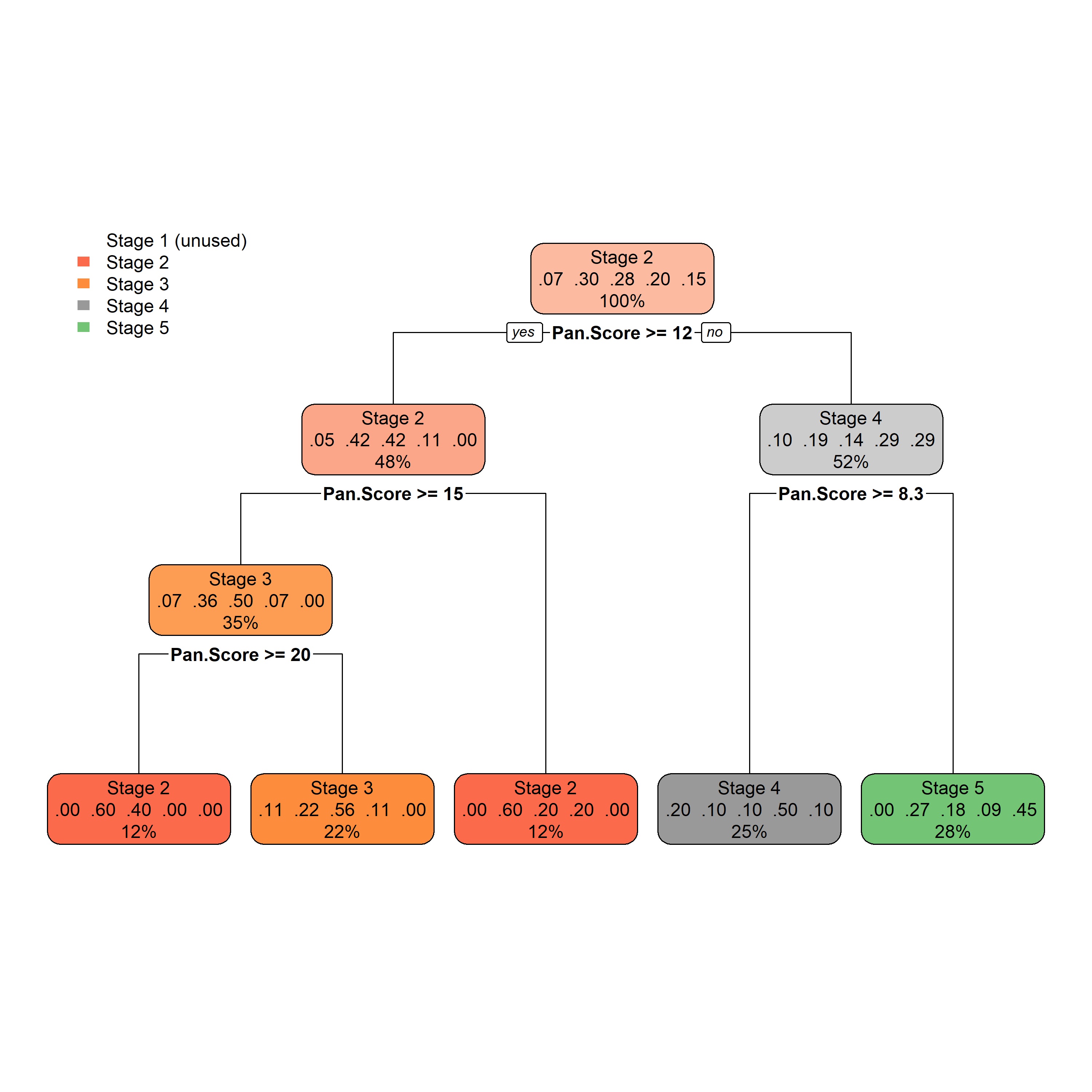

Supplement: Supplementary file 3 [file Image_2.jpeg]
